# Handlebar Hernia: A Rare Type of Abdominal Wall Hernia

**DOI:** 10.4103/1319-3767.37805

**Published:** 2008-01

**Authors:** Khairi A. F. Hassan, Mohamed A. Elsharawy, Khaled Moghazy, Abdulaziz AlQurain

**Affiliations:** Department of Surgery, College of Medicine, King Faisal University, Dammam, Kingdom of Saudi Arabia; * Department of Radiology, College of Medicine, King Faisal University, Dammam, Kingdom of Saudi Arabia; ** Department of Gastroenterology, College of Medicine, King Faisal University, Dammam, Kingdom of Saudi Arabia

**Keywords:** Handlebar, hernia

## Abstract

Handlebar hernias are abdominal wall hernias resulting from direct trauma to the anterior abdominal wall. They usually result at weak anatomic locations of the abdominal wall. Such traumatic hernias are rare, requiring a high index of suspicion for a clinical diagnosis. We report the case of a handlebar hernia resulting from an injury sustained during a vehicular injury, and discuss the management of such injuries.

Abdominal wall hernias caused by direct trauma from handlebar-like objects, and therefore called “handlebar hernias”, are a rare occurrence. We report a case of such hernia associated with significant intraabdominal injury. A 56-year-old male presented with severe blunt abdominal trauma caused by the gear-poke of a car. There was a defect in the anterior abdominal wall associated with mesenteric tear and a gangrenous segment of the ileum. The gangrenous bowel was resected with primary end-to-end anastomosis and the hernia was repaired in layers. A high index of clinical suspicion is required to diagnose such a type of hernia. Traumatic hernia of the anterior abdominal wall is rare; handlebar hernia is an example of traumatic hernia. It was first described by Dimyan *et al.*[1] in 1980, and it was caused by hitting the abdomen against a handlebar-like objects. In literature, less than 30 cases of handlebar hernia have been reported. We report another case associated with significant intraabdominal injury. The aim of this report is to evaluate the clinical presentation and the associated management of such cases.

## CASE REPORT

**Figure 1:**
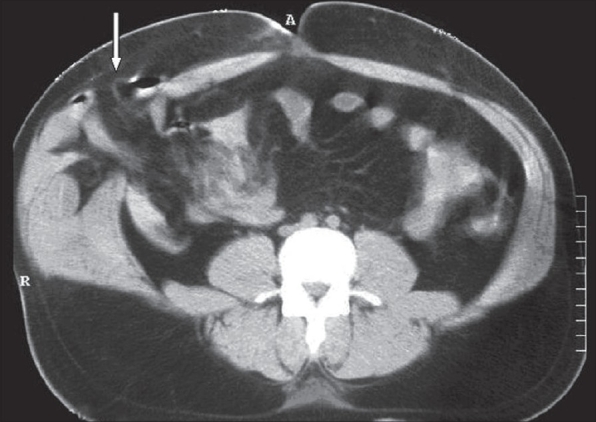
Axial CT of the abdomen: A defect at the anterior abdominal wall muscles (arrow) with bowel loops herniating through it

A 56-year-old male presented to the emergency room 4h after a motor car accident during which his right lower quadrant sustained a direct hit by the gear-poke of a car. There was no history of previous abdominal wall hernias. The vital signs were stable. Abdominal examination revealed tender irreducible soft tissue swelling in the right iliac fossa with superficial ecchymosis and positive cough impulse. Muscular guarding, tenderness and rebound tenderness was observed all over the abdomen. Bowel sounds were absent. There were no associated injuries and the rest of the physical examination did not reveal any abnormality. The laboratory investigations were within the normal limits. The radiological examination of the cervical spine, chest and abdomen were normal. Computed tomography (CT) of the abdomen showed a defect in the anterior abdominal wall muscles in the right iliac fossa with bowel loops herniating through the defect [Figure 1].

Exploratory laparotomy was performed. There was about 200 ml of free blood in the peritoneal cavity and transverse tear of the mid-ileal mesentery causing a gangrene of approximately 20 cm of the bowel. There was also a defect of approximately 8 × 5 cm involving all the layers of the anterior abdominal wall muscles in the right iliac fossa with intact skin. Lacerated muscles were present at the edges of the defect. Active mesenteric bleeding vessels were ligated. The devascularized segment of ileum was resected and end-to-end anastomosis was performed. The mesenteric defect was closed. No other intraabdominal injuries were found. The abdominal wall defect was repaired in layers. The muscular and fascial defects were closed in the anatomic layers. The abdominal incision was closed in a standard manner. Postoperative course was uneventful. Follow up after 2 years showed no evidence of recurrence.

## DISCUSSION

Traumatic abdominal wall hernias are produced by the direct blunt trauma from an object that has insufficient force to penetrate the skin, yet able to disrupt the deeper tissues of muscles and fascia. This is possible because the skin is more elastic than the rest of the layers.[1] There are three major types of traumatic abdominal wall hernias based on the mechanism of injury and the size of the defect. Type I abdominal wall hernia involves a small defect caused by blunt trauma. Type II hernia is a larger defect developed during the high-energy transfer events such as motor vehicle crash or fall from a height. Type III hernias are those defects that involves intraabdominal bowel herniation that has been described for deceleration injuries.[2] Handlebar hernias are often under type I abdominal wall hernias and associated intraabdominal injuries are rare.[1,3,4] Although handlebar injury was the reason for our case, the presentation was typical of type III hernia. The energy from the accident of a high-speed motor car may explain the extent of injury in our patient.

The diagnosis is usually made on the basis of history and physical examination. However, ultrasonography and CT scans may be helpful in difficult cases.[5,6] In rare cases, the hernia would not be identified until exploratory laparoscopy[7] or laparotomy[8] was performed for the associated injuries. Some criteria have been proposed to identify traumatic hernias: absence of preexisting hernia in the same location, evidence of abdominal injury at presentation and immediate or delayed development of the hernia (usually close to the site of injury).[8] In our case, clinical picture and CT findings confirmed the diagnosis.

With the exception of the rare case of thoracic handlebar hernia,[9] all other reported cases were related to the abdominal wall. The abdominal wall hernia is usually found at weak anatomical locations such as the region encompassing the lower lateral abdomen to the rectus sheath. This explains that in the majority of the reported cases of handlebar hernia, including our case, the abdominal wall defect was in the lower abdomen.[2,3] Only two cases were reported for the upper quadrant of the abdomen.[1] The abrupt increase in intraabdominal pressure is responsible for the poor correlation between the site of impact and the resultant defect.[3]

The majority of the cases described with handlebar hernias were caused by low-energy mechanisms such as bicycle or motorcycle. Therefore, in most of the cases, there was no significant intraabdominal injury.[1,4,5] In few cases, including our case, the hernia was caused by high-energy mechanism such as motor-car accidents.[3,10] These cases have been reported with significant intraabdominal injury. The most commonly reported injuries were mesenteric and serosal tears. There are two possible mechanisms of blunt mesenteric injuries: (1) a crushing force applied to the bowel against the spine and (2) shearing forces of the bowel and mesentery along the lines of attachment.[3]

After dealing with all associated intraabdominal injuries, definitive treatment of these hernias mandates surgical exploration and prompt repair to prevent incarceration or strangulation. This repair can be performed with primary closure if the tissue allows or with prosthetic material if the defect is too large.[9,11] Debate exists regarding the local wound exploration *vs*. midline exploratory laparotomy to rule out the intraabdominal injuries.[12] Associated intraabdominal injury, as in our study, would necessitate exploratory laparotomy or extensive local incision. If all the indicators of intraabdominal injury are negative, local wound exploration provides the best anatomic layered repair with subsequent minimal residual defect and improved long-term cosmesis.

In summary, a high level of clinical suspicion for traumatic hernia is required in cases of severe abdominal wall injury. Such patients should be examined and treated properly for the possibility of significant intraabdominal injury.

## References

[1] Dimyan W, Robb J, MacKay C (1980). Handlebar hernia. J Trauma.

[2] Goliath J, Mittal V, McDonough J (2004). Traumatic handlebar hernia: A rare abdominal wall hernia. J Pediatr Surg.

[3] Cheng S, Ko W, Liu C (2005). Handlebar hernia: A misleading term. Injury Extra.

[4] Chen HY, Sheu MH, Tseng LM (2005). Bicycle-handlebar hernia: A rare traumatic abdominal wall hernia. J Chin Med Assoc.

[5] Prada Arias M, Dargallo Carbonell T, Estevez Martinez E, Bautista Casasnovas A, Varela Cives R (2004). Handlebar hernia in children: Two cases and review of the literature. Eur J Pediatr Surg.

[6] Mitchiner JC (1990). Handlebar hernia: Diagnosis by abdominal computed tomography. Ann Emerg Med.

[7] Iinuma Y, Yamazaki Y, Hirose Y, Kinoshita H, Kumagai K, Tanaka T (2005). A case of a traumatic abdominal wall hernia that could not be identified until exploratory laparoscopy was performed. Pediatr Surg Int.

[8] Sahdev P, Garramore RR, Desani B, Ferris V, Welch JP (1992). Traumatic abdominal hernia: Report of three cases and review of the literature. Am J Emerg Med.

[9] Holmes JH, Hall RA, Schaller RT (2002). Thoracic handlebar hernia: Presentation and management. J Trauma.

[10] Huang CW, Nee CH, Juan TK, Pan CK, Ker CG, Juan CC (2004). Handlebar hernia with jejunal and duodenal injuries: A case report. Kaohsiung J Med Sci.

[11] Jones BV, Sanchez JA, Vinh D (1989). Acute traumatic abdominal wall hernia. Am J Emerg Med.

[12] Perez VM, McDonald AD, Ghani A, Bleacher JH (1998). Handlebar hernia: A rare traumatic abdominal wall hernia. J Trauma.

